# Wirkmechanismen antidepressiver Pharmakotherapie: Gehirn und Psyche – Körper und Umwelt

**DOI:** 10.1007/s00115-024-01786-3

**Published:** 2025-01-16

**Authors:** Moritz Spangemacher, Jonathan Reinwald, Hana Adolphi, Laura Kärtner, Lea J. Mertens, Christian N. Schmitz, Gerhard Gründer

**Affiliations:** 1 Abteilung für Molekulares Neuroimaging, Zentralinstitut für Seelische Gesundheit, Medizinische Fakultät Mannheim, Universität Heidelberg, 68159 Mannheim, Deutschland; 2 Klinik für Psychiatrie und Psychotherapie, Zentralinstitut für Seelische Gesundheit, Medizinische Fakultät Mannheim, Universität Heidelberg, Mannheim, Deutschland; 3 Standort Mannheim, Deutsches Zentrum für Psychische Gesundheit (DZPG), Mannheim, Deutschland; 4 AG Translationales Imaging, Klinik für Psychiatrie und Psychotherapie, Zentralinstitut für Seelische Gesundheit, Medizinische Fakultät Mannheim, Universität Heidelberg, Mannheim, Deutschland; 5 AG Systemische Neurowissenschaften und Psychische Gesundheit, Klinik für Psychiatrie und Psychotherapie, Universitätsklinikum Mainz, Johannes-Gutenberg-Universität, Mainz, Deutschland

**Keywords:** Antidepressiva, Extrapharmakologische Faktoren, Umwelt, Kontext, Neuroplastizität, Antidepressants, Extrapharmacological factors, Environment, Context, Neuroplasticity

## Abstract

**Hintergrund:**

Neue antidepressive Substanzen stellen die Erklärungsansätze zu Wirkmechanismen der traditionellen Psychopharmakologie vor Herausforderungen.

**Fragestellung:**

Was könnten gemeinsame Effekte der verschiedenen Antidepressiva sein und welche Rolle spielen dabei jeweils extrapharmakologische Faktoren wie Körper und Umwelt?

**Material und Methode:**

Die verfügbare Literatur über klinische und präklinische Daten zu vermuteten gemeinsamen Wirkfaktoren von serotonergen Psychedelika, (Es‑)Ketamin, monoaminergen Antidepressiva und Zuranolon wird dargestellt und der Einfluss von Kontextfaktoren auf die jeweiligen Wirkmechanismen diskutiert.

**Ergebnisse:**

Es deutet vieles darauf hin, dass klassischere und neuere pharmakologische Ansätze der Depressionsbehandlung ähnliche Wirkmechanismen teilen könnten. Diese Mechanismen begünstigen langfristige Neuroplastizität, die nachgeschaltete molekulare Kaskaden auslösen können und vice versa. Darüber hinaus wurde für die meisten antidepressiven Substanzen auch eine Verbesserung der negativen Verzerrung in der emotionalen Verarbeitung nachgewiesen. Der Einfluss extrapharmakologischer Faktoren scheint notwendig zu sein, damit die biopsychologischen Veränderungen antidepressiv wirksam sein können.

**Schlussfolgerungen:**

Anstatt Faktoren wie Umwelt, Körper und soziale Interaktionen zu den Placeboeffekten zu zählen, sollten sie als essenzieller Baustein der antidepressiven Wirkung geprüft und in der klinischen Versorgung mitbedacht werden.

## Abstract (en)

**Background:**

Novel antidepressive substances are challenging the explanations for the mechanisms of action of traditional psychopharmacology.

**Objective:**

What could be the shared effects of various antidepressants and in this context what role do extrapharmacological factors, such as the body and environment, play?

**Material and method:**

The available literature on clinical and preclinical data for assumed combined active factors of serotonergic psychedelic drugs, (es)ketamine, monoaminergic antidepressants and zuranolone are presented and the influence of context factors on the individual mechanisms of action is discussed.

**Results:**

There are many indications that classical and novel pharmacological approaches could share similar mechanisms of action in the treatment of depression. These mechanisms favor long-term neuroplasticity, which can trigger subsequent molecular cascades and vice versa. Furthermore, an improvement in the negative bias in emotional processing could be detected for most antidepressive substances. The influence of extrapharmacological factors appears to be necessary so that the biopsychological alterations can have an antidepressive effect.

**Conclusion:**

Instead of attributing factors such as environment, body and social interaction to placebo effects, they should be tested as essential components of the antidepressive effect and considered in the clinical practice.

## Hintergrund

Neue pharmakologische Ansätze wie die klassischen serotonergen Psychedelika, das Neurosteroid Zuranolon, aber auch nichtpharmakologische Stimulationsverfahren wie die repetitive transkranielle Magnetstimulation (rTMS) werfen grundlegende Fragen zu den allgemeinen Wirkfaktoren antidepressiver Interventionen auf [28].

Einerseits haben sich Erklärungsansätze von monokausalen Konzepten, die auf molekularen Dysfunktionen einzelner Neurotransmitter basieren, zunehmend entfernt. Im Vordergrund steht aktuell ein komplexeres Erklärungsmodell, das vor allem die Modulationsfähigkeit der Neuronen auf zellulärer Ebene und ihre Interaktion innerhalb von Gehirnnetzwerken auf struktureller und funktioneller Ebene betrachtet.

Andererseits stellen bewusstseinsverändernde Substanzen wie Psilocybin, aber auch (Es‑)Ketamin, die Rolle des Kontexts wieder mehr in den Mittelpunkt der Behandlung. Daraus ergibt sich jedoch auch eine Herausforderung für die wissenschaftliche Prüfung dieser Substanzen, da Umwelt- und Kontextfaktoren kaum oder nur sehr schwer von Placeboeffekten abzugrenzen sind. Wie kann man die kontextuelle Einbettung der Behandlung am besten kontrollieren und standardisieren [37]?

Dabei handelt es sich jedoch generell nicht um neue Fragestellungen, sondern um inhärente Grundprinzipien der Psychopharmakologie, die die Erforschung psychotroper Substanzen, die therapeutisch eingesetzt werden, seit jeher begleiten. Der Schweizer Roland Kuhn (1912–2005), der das erste klassische Antidepressivum Imipramin entdeckte, kritisierte scharf die Untersuchung antidepressiver Medikamente unter artifiziellen, „vollständig kontrollierten Lebensbedingungen“ ohne die Beachtung der Umwelt und sozialen Umgebung des Individuums. Trotz seines stark biologisch geprägten Bildes der damals noch als „endogen“ bezeichneten Depression plädierte er gegen eine zu starke Standardisierung des Kontexts der Behandlung.

„Die klinische Erfahrung lehrt …, dass die psychopathologischen Symptome weitgehend eben durch ‚Interaktion‘ entstehen, sie sind wie der angemessene Ausdruck lautet: ‚Tatsachen der Verständigung‘“ [24].

Kuhn stellte fest, dass sich Psychopathologie nicht nur im Gehirn entfalte und deswegen auch der Kontext beachtet werden müsse, wenn wir Medikamente verschreiben und auf ihre Wirksamkeit prüfen. Hier zeigt sie sich eine lange Tradition in der Erforschung antidepressiver Behandlungen und ihrer Interaktion zwischen Gehirn, Psyche, Körper und Umwelt. Diese vier Entitäten können epistemologisch nicht voneinander getrennt werden und stehen in ständig dynamischer Wechselwirkung miteinander. Für den Zweck dieses Artikels bieten sie jedoch ein geeignetes Konstrukt, um die verschiedenen Ebenen, auf denen Antidepressiva wirken, besser beschreiben zu können.

Im Folgenden werden nach dieser Einteilung die gemeinsamen biopsychologischen Wirkmechanismen klassischer Antidepressiva (wie z. B. Serotoninrückaufnahmehemmer [SSRI]) und neuerer, emergenter Substanzen wie Psychedelika beschrieben sowie deren Interaktion mit extrapharmakologischen Umwelt- und Kontextfaktoren herausgearbeitet.

## Gehirn und Psyche

Aktuell wird in der Literatur vermehrt die Heterogenität der Depression und ihrer Genese als multifaktorielle Erkrankung betont. Zunehmend können dabei auch neurobiologisch distinkte Subtypen beschrieben werden [39]. Obwohl die Symptomatik bei jedem/r Patient:in unterschiedlich ist, zeigen sich gleichzeitig auch viele Gemeinsamkeiten im Bereich biologischer Veränderungen sowie bei Defiziten auf der Verhaltens- und Gefühlsebene [33].

Diese Veränderungen führen letztlich auf einer gemeinsamen Endstrecke zu einer sich selbst verstärkenden, anhaltenden Negativspirale, die klinisch in einen depressiven Phänotyp mündet. In der antidepressiven Behandlung sollten sie gezielt adressiert und, wenn möglich, rückgängig gemacht werden.

Pathologische Modifikationen finden auf Ebene der Zellen und Zellverbindungen, in der Balance der Neurotransmitter sowie auf der Ebene der Gehirnnetzwerke statt. Dabei handelt es sich meist um Veränderungen von Gehirnnetzwerken, die emotionale und kognitive Informationen verarbeiten und unter anderem Funktionen wie Energieniveau oder soziale Kompetenzen regulieren [33].

Auf zellulärer Ebene konnten Funktionsstörungen der Synapsen bei depressiven Patient:innen und in Tiermodellen der Depression nachgewiesen werden [13, 25]. Pathologische Prozesse an den Verbindungsstellen zwischen Neuronen, an denen elektrische Signale und chemische Neurotransmitter vom Axon eines präsynaptischen Neurons zu den Dendriten eines postsynaptischen Neurons gelangen, führen langfristig zu neuronaler Atrophie und Zelltod [13]. Eine Vielzahl von Studien deutet darauf hin, dass die Behandlung von Depressionen auch über die Verbesserung von Neuroplastizität, Synapto- und Neurogenese vermittelt wird, d. h. über die Umschaltung dysfunktionaler Schaltkreise und dem Wachstum neuer Nervenendigungen und Synapsen im Gehirn [7, 23, 36].

In mehreren präklinischen und klinischen Untersuchungen konnte eine generelle Verstärkung der Neuroplastizität durch verschiedene Antidepressiva und eine Vermehrung der Synapsendichte gezeigt werden [7, 23, 36]. Auch wenn Zeitrahmen und Ausmaß des Effekts zu variieren scheinen, scheint ein gemeinsamer Mechanismus die direkte Bindung an die Tropomyosin-Rezeptor-Kinase B (TrkB) zu sein, an die normalerweise das Neurotrophin BDNF („brain-derived neurotrophic factor“) bindet und diese aktiviert [8, 29]. Die Aktivierung von TrkB, die sich auf der Zelloberfläche der Neuronen befindet, löst mehrere intrazelluläre Signalkaskaden aus, die Zelldifferenzierung und Zellwachstum fördern können [14]. Wie genau die Auswirkungen einer optimierten Neuroplastizität aussehen, hängt jedoch stark von der Lokalisation im Gehirn ab.

Auf struktureller Ebene sind die Verhaltenssymptome der Depression mit Veränderungen des Volumens, der Aktivität und der Konnektivität zerebraler Netzwerke assoziiert, die an der emotionalen Wahrnehmung, der Belohnungsverarbeitung, der Motivation und den exekutiven Funktionen beteiligt sind. Auch wenn eine ausgeprägte methodologische Heterogenität in der Literatur eine Aussage über konvergente Mechanismen erschwert, scheinen eine Veränderung der Konnektivität zwischen präfrontalem Kortex und Hippokampus, Reduktion der Aktivität des sog. Default Mode Networks sowie Interaktionen mit dem limbischen System relevant [39].

Die synaptische Dysfunktion zeigt sich am deutlichsten in Pyramidenneuronen, einer weit verbreiteten Klasse von Neuronen, die Glutamat freisetzen und dadurch Zielneurone aktivieren. Diese setzen dann andere Neurotransmitter wie Serotonin, Noradrenalin, Dopamin und Acetylcholin frei. Eine verbesserte Synaptogenese könnte hier zu einer Optimierung der Freisetzung dieser Neurotransmitter führen. Die Ausschüttung von Serotonin und vor allem Glutamat löst wiederum eine nachgeschaltete Signalkaskade aus, die essenziell für Neuroplastizität ist, sodass man hier vermutlich von einem sich selbst verstärkenden Mechanismus sprechen kann [2].

Während die Serotoninmangelhypothese in der Ätiologie der Depression weiterhin kontrovers diskutiert wird [21, 30], ist eine direkte Wirkung von Antidepressiva auf Neurotransmitter unbestreitbar [20]. In den meisten Positronenemissionstomographie(PET)-Studien mit SSRI ist die antidepressive Wirkung mit einer mindestens 80 %igen Besetzung des Serotonintransporters (SERT), wie sie mit den Standarddosierungen dieser Substanzen erreicht wird, assoziiert. Bei noradrenergen Substanzen scheint eine 50 %ige Besetzung des Noradrenalintransporters (NAT) ausreichend zu sein [20]. Nur für den Noradrenalin-Dopamin-Rückaufnahmehemmer (NDRI) Bupropion und die Monooxidase(MAO-A)-Hemmer Moclobemid und Tranylcypromin konnten keine Schwellenwerte für die Bindung an ihre molekulare Targets gefunden werden [20].

Auch in PET-Studien mit dem partiellen Serotoninagonisten Psilocybin bei gesunden Probanden korrelierte die Konzentration des Metaboliten Psilocin mit der Besetzung des 5HT_2A_-Serotoninrezeptors und der Intensität der akuten subjektiven Erfahrung [27]. Diese wiederum korrelierte in anderen Studien mit dem therapeutischen Ansprechen auf das klassische Psychedelikum [41].

Für den N‑Methyl-D-Aspartat(NMDA)-Antagonisten Ketamin ist die Situation insgesamt etwas komplexer. Auch wenn Ketamin ebenfalls Auswirkungen auf Neurotransmitter wie Serotonin hat, vermutet man primär einen glutamatergen Mechanismus über die Veränderung der exzitatorisch-inhibitorischen Dysbalance [2]. Dass hierbei nicht nur ein Defizit an glutamaterg-exzitatorischen Prozessen eine Rolle spielt, wird auch dadurch gezeigt, dass das ebenfalls antidepressiv wirksame Zuranolon hauptsächlich modulierend über den inhibitorischen γ‑Aminobuttersäure(GABA)-Rezeptor wirkt [11]. Auch hier scheint es sich um ein komplexes Gleichgewicht zu handeln, das in wechselseitiger Interaktion mit neuroplastischen Prozessen steht.

Ein erhöhtes bzw. schnelleres Zellwachstum wird häufig mit einer erhöhten kognitiven Flexibilität assoziiert, die es den Patient:innen ermöglicht, sich leichter von pathologischen Gedanken, Gefühlen und Verhaltensweisen zu distanzieren [35]. Insbesondere das Feststecken in einem Teufelskreis der negativen Informationsverarbeitung bei der Depression kann als Beeinträchtigung in der psychologischen Flexibilität verstanden werden. In diesem Zusammenhang wurde bereits vermutet, dass eine erhöhte Neuroplastizität die emotionale und kognitive Verarbeitung verbessert, indem Wahrnehmung, Aufmerksamkeit und Erinnerung von negativen zu positiven affektiven Ereignissen verschoben werden [19]. Die Verabreichung von Antidepressiva förderte bei Patient:innen mit Depressionen die Erkennung glücklicher Gesichtsausdrücke und den Abruf positiver gegenüber negativer selbstbezogener Erinnerungen [19]. Die Verbesserung dieser negativen Verzerrung konnte für mehrere antidepressive Substanzen nachgewiesen werden [5, 19]. Sie zeigt sich trotz unterschiedlicher Therapieparadigmata (z. B. chronische Verabreichung von SSRI, wiederholte Gaben von Ketamininfusionen, Einmalgabe von Psychedelika) bereits früh in der Behandlung. Es könnte sich hierbei um einen Marker für gesteigerte Neuroplastizität und Therapieansprechen handeln [6].

Zusammenfassend resultieren gemeinsame Mechanismen neuerer pharmakologischer antidepressiver Interventionen wie Ketamin und serotonerge Psychedelika und monoaminbasierte Therapieansätze wie SSRI vermutlich aus der Induktion nachgeschalteter molekularer Kaskaden, die zu einer länger anhaltenden Neuroplastizität führen. Zudem bewirken sie akutere Veränderungen der emotionalen Verarbeitung und des Verhaltens, die im Sinne einer positiven Lernerfahrung affirmative Valenzen fördern können.

## Körper und Umwelt

Gemäß dem Psychiater und Phänomenologen Thomas Fuchs entsteht Psychopathologie erst in der Interaktion zwischen Leib, Raum und Intersubjektivität [15]. Auch in der gelebten Erfahrung der Depression und ihrer Behandlung zeigen sich hier mehrere Beispiele [16]: Negative kognitive Verzerrungen können erst im Kontext neuer Lernerfahrungen im Alltag verändert werden. Verhaltensänderungen sind oftmals nur persistent, wenn sie von einem sozialen Netz nachhaltig gefördert werden [19]. Eine Reduktion von Anhedonie und Antriebslosigkeit ist wenig wirksam, wenn chronische Schmerzen das körperliche Wohlbefinden weiterhin beeinträchtigen.

Auch die Erhöhung der Neuroplastizität kann positive sowie negative Konsequenzen haben [36]. In einer präklinischen Studie konnte gezeigt werden, dass die Gabe eines SSRI in einer förderlichen Umwelt zwar antidepressive Effekte hatte, in einer stressbezogenen Umgebung den depressiven Phänotyp sogar verschlechterte [1]. Zudem resultierte soziale Isolation in einer Aufhebung der antidepressiven Effekte von Fluoxetin und Sertralin [12, 42]. Auch spezifischere Umweltfaktoren wie z. B. das Geschlecht der versuchsleitenden Person hatte bei Mäusen einen Einfluss auf das antidepressive Ansprechen, z. B. bei der Behandlung mit Ketamin [17].

Interessanterweise wurden Umweltfaktoren konzeptionell schon früh in der Erforschung der Behandlung mit Psychedelika in Betracht gezogen. So spricht man hier von „Set und Setting“ als essenziellem Wirkfaktor, der von der Wirkung der Substanz nicht epistemologisch abgegrenzt werden kann [18]. Dabei steht „Set“ für die aktuelle psychische und körperliche Verfassung der Person und „Setting“ für die Umweltkonstellation, in der die Substanz verabreicht wird. Bis heute wird spezifisch darauf geachtet, dass Psychedelika in einem besonders eingerichteten Therapieraum in Anwesenheit von zwei Therapeut:innen gegeben werden, um eine ruhige, angenehme Atmosphäre zu schaffen [18]. Dabei zeigte sich in mehreren Studien, dass die Ausprägung der therapeutischen Verbindung, die die/der Patient:in mit dem Therapeutenteam aufgebaut hatte, mit dem letztendlichen Therapieresultat korrelierte [26, 31]. Auch wenn Psychedelika eine besondere Sensitivität für die Umwelt zu kreieren scheinen, ist es fraglich, warum die gleichen Mechanismen nicht auch für andere neuroplastizitätsfördernde Antidepressiva gelten sollten. Bezüglich der erwähnten therapeutischen Allianz konnte dies bereits nachgewiesen werden. Die therapeutische Bindung ist nicht nur nachweislich schulenübergreifend einer der wesentlichen Wirkfaktoren in der Psychotherapie [3], sondern konnte auch bei der pharmakotherapeutischen Gabe von Antidepressiva das Behandlungsergebnis prädizieren [9, 43]. Daraus ergeben sich einige Implikation nicht nur für die Erforschung, sondern auch für die klinische Verabreichung von Antidepressiva wie SSRI [37]. Zum Beispiel könnte eine gezielte Planung sozialer Interaktionen nach Beginn der Medikation sowie eine Optimierung des meist rudimentären Komforts auf psychiatrischen Stationen daraus abgeleitet werden [37].

Auch der Körper, in dem die neuroplastischen Effekte stattfinden, kann als Teil des Kontexts verstanden werden. Antidepressiva wirken nicht nur zentral, sondern haben auch direkte periphere Effekte, wie unter anderem neue Untersuchungen am Darmmikrobiom gezeigt haben [8]. Entzündliche Prozesse in und außerhalb des zentralen Nervensystems beeinflussen die Wirksamkeit von Antidepressiva [34]. Im Sinne einer bidirektionalen Beziehung haben Antidepressiva gleichzeitig entzündungshemmende Wirkungen [4, 34]. Ein gesunder Körper ist aus dieser Perspektive essenziell für die Remission unter antidepressiven Substanzen [22]. Nebenwirkungen von Antidepressiva, die häufig das Risiko eines metabolischen Syndroms begünstigen, sollten unter diesem Aspekt besonders kritisch gesehen werden [22].

Generell scheint eine einfache Erhöhung der Neuroplastizität nicht ausreichend für die Erklärung des antidepressiven Effekts der vorbeschriebenen Substanzen. So führt beispielsweise auch die eher depressogene psychoaktive Substanz Kokain zu einer Hyperplastizität [32]. Möglicherweise können antidepressive Effekte besser mit dem Konzept der „Metaplastizität“ erklärt werden [32, 40]. Metaplastizität bezeichnet die Fähigkeit von Neuronen, die Anforderungen für die Induktion synaptischer Plastizität basierend auf ihrer vorherigen Aktivität anzupassen. Kurz gesagt beschreibt sie, wie leicht oder schwer es für eine Synapse ist, sich plastisch zu verändern, abhängig von früheren Erfahrungen oder Aktivitäten. Dies bedeutet, dass das Nervensystem seine Plastizität dynamisch anpasst, um eine optimale Balance zwischen Stabilität und Flexibilität aufrechtzuerhalten [40]. Serotonerge Psychedelika und auch Ketamin scheinen im Tier Metaplastizität zu induzieren [32]. Im Speziellen führen sie dazu, dass kritische Lernperioden, die z. B. essenziell für die Entwicklung sozialer Kompetenzen sind, wieder geöffnet werden. Früh erlernte kognitive und emotionale Mechanismen können in diesem Sinne „neu erlernt“ werden [32].

Eine neuere Theorie beschreibt dementsprechend, dass die Wirksamkeit von Psychedelika auf einer Form der „Meta-Kontrolle“ beruht. Dabei würden sie Kognitionen nicht unidirektional in „divergentes vs. konvergentes Denken“ oder „Geschwindigkeit vs. Genauigkeit“ lenken. Vielmehr könnten sie ermöglichen, flexibel und kontextbedingt zwischen den Konditionen zu entscheiden, um eine möglichst balancierte Kognitions- und Affektregulation zu erreichen [38].

## Schlussfolgerung

**Abb. 1:**
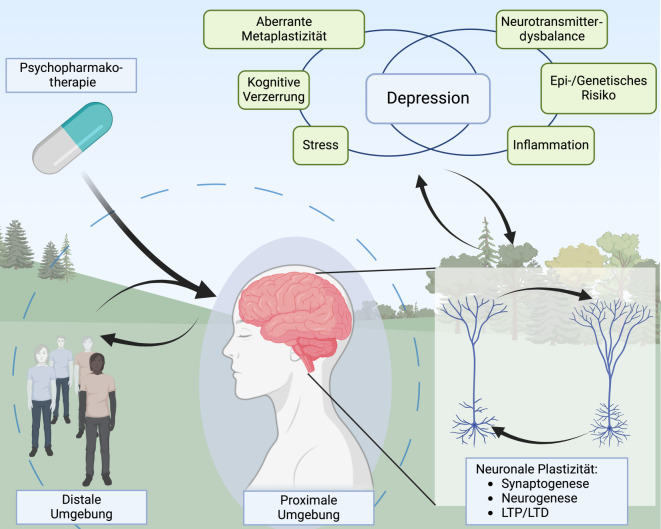
Menschen interagieren sowohl mit ihrer unmittelbaren (proximalen) als auch mit ihrer entfernten (distalen) Umgebung, was sich direkt auf Hirnprozesse auswirkt. Diese Interaktionen beeinflussen die neuronale Plastizität, die durch Synaptogenese (Bildung neuer Synapsen), Neurogenese (Bildung neuer Neuronen) und funktionelle Veränderungen wie Langzeitpotenzierung (LTP) und -depression (LTD) gekennzeichnet ist. Bei psychischen Störungen wie Depressionen, die durch immunologische Prozesse, Neurotransmitterveränderungen, kognitive Verzerrungen, Stress, genetische oder epigenetische Faktoren sowie aberrante Metaplastizität verursacht sein können, wird die neuronale Plastizität oft beeinträchtigt und vice versa. Die Behandlung mit konventionellen und neuen Antidepressiva wirkt auf diesen Kreislauf ein und hat mit der Umgebung sowie den neuropsychologischen Prozessen wechselseitige Interaktionen. (Erstellt mit BioRender.com)

Die Wirkmechanismen antidepressiver Substanzen, wie Neuro- und Metaplastizität, die Veränderung negativer kognitiver Verzerrungen und die Stabilisierung von Neurotransmitterdysbalancen, hängen stark von Interaktionen mit der Umwelt, anderen Menschen und dem eigenen Körper ab (Abb. 1). Anstatt diese Kontextfaktoren grob als Placeboeffekte einzuordnen, sollten sie in der präklinischen und klinischen Forschung sowie in der Versorgung stärker berücksichtigt und untersucht werden.

Die Rückschlüsse, die sich bereits jetzt in den klinischen Alltag übertragen lassen, implizieren, dass eine Verschreibung eines Antidepressivums nie unabhängig vom Kontext des/der Betroffenen geschehen sollte. Gerade bei Patient:innen, die sich in einer prekären sozialen Situation befinden oder mit Einsamkeit hadern, sollte die Gabe therapeutisch multimodal eingebettet sein und auf eine stabile therapeutische Beziehung geachtet werden.

Bei ambulanten Erstkontakten z. B. ist es demnach wichtig, ein sozialdienstliches und psychotherapeutisches Therapiekonzept zu planen, was aktuell leider nicht der Versorgungsrealität entspricht. Zudem benötigt es eine engere Verzahnung von Psychotherapie und Pharmakotherapie sowohl stationär als auch ambulant. Ansonsten besteht das Risiko, dass die ungerichtete Wirkung der antidepressiven Substanz auch negative Auswirkungen für das Individuum haben kann.

Zudem könnten gemeinsame Konzepte der Psycho- und Pharmakotherapie ausgearbeitet werden, die bis jetzt leider noch kaum bestehen. So könnte im Sinne einer prozessorientierten Psychotherapie für eine Patient:innenkohorte die psychologische Flexibilität gesteigert werden, während sich für eine andere Kohorte psychopharmakologisch mehr eine Behandlung zur Förderung der Emotionsregulation anbieten könnte.

Letztendlich lässt sich auch das entfernte kontextuelle Umfeld nicht von der antidepressiven Wirkung einer Substanz trennen – weder bei der klassischen Pharmakotherapie noch bei neueren Substanzen wie Psychedelika oder Ketamin. Welche Auswirkungen in diesem Sinne Sozioökonomie und Klima haben, sollte in Zukunft mehr erforscht werden.

## Fazit für die Praxis

- Neue antidepressive Pharmakotherapien wie (Es‑)Ketamin und klassischere Therapieansätze wie SSRI teilen gemeinsame Wirkmechanismen.
- Dazu zählen die Erhöhung der Neuroplastizität durch direkte Bindung am Tropomyosin-Rezeptor-Kinase B(TrkB)-Rezeptor, die Verbesserung der emotionalen und kognitiven Verarbeitung depressiver Wahrnehmungen sowie ein direkter Effekt auf molekulare Neurotransmitter.
- Eine einfache Erhöhung der Neuroplastizität ist für die antidepressive Wirkung nicht ausreichend. Die Wirkung wird maßgeblich von Umweltfaktoren mitbestimmt und lässt sich besser durch das Konzept der Metaplastizität erklären.
- Kontextfaktoren wie soziale Interaktionen, körperliche Verfassung und Umweltbedingungen sollten bei der Verabreichung von Antidepressiva in klinischen Studien und in der Versorgung generell mehr beachtet werden.
- Unsere Rückschlüsse fordern eine engere Verzahnung von Psychotherapie und Pharmakotherapie sowohl im stationären als auch ambulanten Setting.
